# Global host metabolic response to *Plasmodium vivax *infection: a ^1^H NMR based urinary metabonomic study

**DOI:** 10.1186/1475-2875-10-384

**Published:** 2011-12-23

**Authors:** Arjun Sengupta, Soumita Ghosh, Angika Basant, Suhas Malusare, Parul Johri, Sulabha Pathak, Shobhona Sharma, Haripalsingh M Sonawat

**Affiliations:** 1 Department of Chemical Sciences, Tata Institute of Fundamental Research, Homi Bhabha Road, Mumbai 400 005, India; 2 Department of Biological Sciences, Tata Institute of Fundamental Research, Homi Bhabha Road, Mumbai 400 005, India; 3 The Division of Biological Sciences, University of Chicago, 5812, S. Ellis Avenue, Chicago, IL 60637, USA

**Keywords:** *Plasmodium vivax*, NMR, metabonomics, metabolites, biomarker

## Abstract

**Background:**

*Plasmodium vivax *is responsible for the majority of malarial infection in the Indian subcontinent. This species of the parasite is generally believed to cause a relatively benign form of the disease. However, recent reports from different parts of the world indicate that vivax malaria can also have severe manifestation. Host response to the parasite invasion is thought to be an important factor in determining the severity of manifestation. In this paper, attempt was made to determine the host metabolic response associated with *P. vivax *infection by means of NMR spectroscopy-based metabonomic techniques in an attempt to better understand the disease pathology.

**Methods:**

NMR spectroscopy of urine samples from *P. vivax-*infected patients, healthy individuals and non-malarial fever patients were carried out followed by multivariate statistical analysis. Two data analysis techniques were employed, namely, Principal Component Analysis [PCA] and Orthogonal Projection to Latent Structure Discriminant Analysis [OPLS-DA]. Several NMR signals from the urinary metabolites were further selected for univariate comparison among the classes.

**Results:**

The urine metabolic profiles of *P. vivax-*infected patients were distinct from those of healthy individuals as well as of non-malarial fever patients. A highly predictive model was constructed from urine profile of malarial and non-malarial fever patients. Several metabolites were found to be varying significantly across these cohorts. Urinary ornithine seems to have the potential to be used as biomarkers of vivax malaria. An increasing trend in pipecolic acid was also observed. The results suggest impairment in the functioning of liver as well as impairment in urea cycle.

**Conclusions:**

The results open up a possibility of non-invasive analysis and diagnosis of *P. vivax *using urine metabolic profile. Distinct variations in certain metabolites were recorded, and amongst these, ornithine may have the potential of being used as biomarker of malaria. Pipecolic acid also showed increasing trend in the malaria patient compared to the other groups.

## Background

Malaria is caused by parasites of the genus *Plasmodium*. The five *Plasmodium *species that are responsible for human malaria are *Plasmodium vivax*, *Plasmodium falciparum*, *Plasmodium malariae*, *Plasmodium ovale *and *Plasmodium knowlesi *[1]. Every year, 200-300 million people are affected with malaria with an annual mortality rate of nearly one million [2]. Sub-Saharan Africa and Southeast Asia are some of the most affected regions. In India, *P. vivax *is the predominant cause of clinical malaria [3].

Metabonomics is a comparatively recently developed technology defined as the global, dynamic response of living organism towards genetic and environmental perturbations [4]. The technique involves the NMR or mass spectra analysis of biofluids such as urine and serum, etc. followed by multivariate analyses using Principal Component Analysis [PCA] or Orthogonal Partial Least Square - Discriminant Analysis [OPLSDA]. Essentially this provides the clustering of the samples into classes. This also provides the identity of specific NMR/mass spectral signature[s] that are responsible for the clustering/classification. This, in turn, leads to identification of the metabolite[s] that are specifically perturbed in response to the stress factor [genetic or environmental] under investigation [5]. Metabonomics, although a relatively new technology, is being used extensively in pharmacological industry [6,7]. Metabonomic analysis is also being utilized in identification of novel biomarkers and/or metabolic characterization during different diseases, such as diabetes [8] and congenital heart disease [9].

Malaria is an ancient infectious disease that has afflicted humans since pre-historic times. Severity in the clinical malarial disease occurs frequently, and has been well documented for *P. falciparum *infections [10,11]. The severity or pathogenicity is likely to be due to metabolic complications arising as a result of host parasite interactions in which the pathogen may divert the host nutrients, and/or release toxic metabolites. Metabolomic analysis will allow a direct read out for such complications. Using axenic cultures, the changes in metabolomic profiles have been documented for the intraerythrocytic stages of *P. falciparum *[12-14]. However, there have been very few studies on the effects on the metabolic profile of the host during malarial infection. Although body fluids such as urine and plasma are amenable to such metabolomic analysis, very few reports exist of such studies. Urine, as an easily available fluid, is also a very good reporter of the overall metabolic status of the whole organism. Systemic level analysis of host metabolic response towards malaria is delineated only in two rodent model studies. In one of the studies, existence of sexual dimorphism was shown in the alterations of sera, urine and brain metabolic profile in the rodent model of malaria [15]. In another study Nicholson and co-workers delineated the global metabolic response to *Plasmodium berghei *infection [16]. No metabolomic information exists so far for human patient samples. In particular, very little is known for *P. vivax *patients, although it has been observed recently that *P. vivax *can cause high levels of pathological complications [17,18].

In this report, a NMR based metabonomic approach is delinated to study the urine samples of *P. vivax *malaria patients and try to correlate the changes observed in them to the known and reported biochemical processes. The paper describes a study of global host metabolic responses towards *P. vivax *infection. The results ascertain differences in metabolic response in the urinary metabolic profile of the *P. vivax *infected persons with respect to that of healthy individuals and also with the patients of non-malarial fever. The differential metabolites are delineated, raising the possibility of a non-invasive diagnosis of malaria.

## Methods

### Ethical aspects

This study was approved by and carried out according to the guidelines of the Local Institutional Ethics Committee. Informed consent was obtained prior to sample collection.

### Sample collection

Midstream urine samples (~10 ml) of malaria-infected individuals were collected from local pathological laboratories in sterile tubes containing a final concentration of 0.02% sodium azide. The inclusion criteria followed to recruit the subjects in the study were as follows- (1) Adult males (age ranging from 30 to 50 years), (2) Malaria cohort of those patients that tested positive for *P. vivax *by blood smear microscopy and (3) Subjects able to consent through informed consent document. Certain exclusion criteria were also followed viz. (1) All females and males < 30 and > 50 years of age, (2) Patients with *P. falciparum *and mixed *P. falciparum/P. vivax *infections and (3) Patients with other chronic diseases and/or previous history of any type of malarial infection. In total 53 individuals (all males) were recruited for the study: 21 *P. vivax-*infected patients, 21 healthy controls and 12 patients with non-malarial fever. Their age ranges were: malaria patients: 35 ± 3.95 years: healthy individuals: 44 ± 3.18 years: and non-malarial fever patients: 40.5 ± 5.33 years (median ± standard error). The samples were transported from the site of collection to the laboratory at 0°C and were immediately processed [19].

### Sample preparation for NMR experiments

An aliquot (800 μL) of collected urine sample was mixed with 400 μL phosphate buffer (an 81:19 (v/v) mixture of 0.2 M Na_2_HPO_4 _and 0.2 M NaH_2_PO_4_; pH 7.4, made in deionized and 0.22 mm filtered water). This mixture was homogenized and left to stand for 5 min at ambient temperature following which it was centrifuged for 6 min at 6000 g to remove any suspended matter. The clear supernatant (600 μL) was transferred to 5 mm NMR tube (Wilmad, USA) and 50 μL D_2_O, containing 2, 2-dimethyl-2-silapentane-5-sulfonic acid (DSS) at 0.01% final concentration, was added to it. D_2_O served as a field-frequency lock and DSS was the internal chemical shift reference. The samples were stored frozen at -20°C until required for NMR experiments.

### NMR experiments

The NMR spectra were acquired on AVANCE 500 MHz Bruker NMR spectrometer equipped with a 5 mm broad-band inverse probe. The operating frequency on this machine was 500.13 MHz for ^1^H. For the 1D experiments the spectral width was 12.01 ppm, an excitation pulse of 9 μs (~70°), and a relaxation delay of 1 s between consecutive pulses. This resulted in an acquisition time of 0.68 s and a cycle time of 1.68 s. A total of 64 transients were acquired into 8,192 data points. The water suppression routine involved excitation sculpting using gradients. For processing the FIDs were subjected to exponential multiplication leading to an additional line broadening of 0.20 Hz. A sine bell apodization was also used prior to Fourier transformation. The ^1^H NMR spectra were manually phase and baseline corrected. For identification of metabolites two dimensional (2D) experiments were performed on selected samples. 2D COSY spectra were acquired with 32 transients in the direct dimension for each of the 256 increments in the indirect dimension. The spectral width was 12.01 in both the dimensions. The pulse width used was 11.80 μs with a delay of 1.5 s. The processing parameters of the data included a QSINE window function, exponential multiplication of 0.20 and 0.30 Hz in the direct and indirect dimension respectively, followed by Fourier transformation in both the dimensions. The 2D TOCSY experiments were performed using similar acquisition parameters. The processing parameters were also identical with an extra Gaussian multiplication of 0.10 Hz prior to Fourier transformation in both the dimensions. The 2D spectra aided in assignment of the NMR resonances to the specific metabolites. In addition, comparison with reported experimental and calculated spectra was done using Human Metabolome Data Base (http://www.hmdb.ca/search/spectra).

### Data reduction and analyses

The frequency domain ^1^H NMR spectra were manually phased and baseline corrected. For data reduction the individual spectrum (0.50 to 9.50 ppm) were divided into frequency bins of width 0.04 ppm each resulting in a total of 191 bins per spectrum. Each bin was integrated using MestReC 4.7.0. In order to avoid the artefacts of water suppression and the variable concentration of urea, the region 4.3 to 6.6 ppm was excluded from the binning procedure. The integrals were normalized to the total intensity of the spectrum to compensate for the inter-sample dilution effects. This was achieved by an algorithm developed in-house. The data was mean-centered (the average value of each bin for all samples was subtracted from individual integrals) and Pareto scaled. The preceding operation resulted in similar weight being given to all the bins and the resultant multivariate models were not dominated by bins representing metabolites high in concentration. Next, the data so generated served as input for PCA and OPLS-DA, which were performed on the SIMCA-P + 12.0 platform (Umetrics, Umea, Sweden).

PCA is an unsupervised pattern recognition technique where no class identity is assigned to the samples *a priori*. This helps in visualizing any pattern that is present in the data and also identifies the outliers. The class specific segregation can be refined using OPLS-DA. This is a supervised technique where class specificity is assigned towards the sample set *a priori*. This aided in the visualization of the data as scores plot. In such a plot each point represents a spectrum and the pattern and the segregation in the data can be visualized. This pattern is due to one or more spectral bin(s) which can be visualized using a loadings plot where each point represents a spectral bin. The OPLS-DA model is judged using two parameters. R^2^X signifies the total explained variation in the data whereas the Q^2^(cum) explains the extent of separation between the classes as well as the predictability of the model.

### Integration of NMR peaks

The spectral bins contributing to the variation seen in the OPLS-DA models (visualized in terms of scores plot) were assigned to specific metabolites as described in the previous section. For further quantification of these metabolites, univariate analysis of the NMR spectral peaks was performed in the following way: the peak of the metabolite contributing to the variation in the OPLS-DA model was assigned in the one-dimensional NMR spectral profiles of the individuals. These peaks were integrated, normalized to the whole region of the spectral profile and compared between the classes concerned using student's *t*-test to judge the significance in their difference across the classes.

## Results

### Multivariate statistical analysis

**Figure 1:**
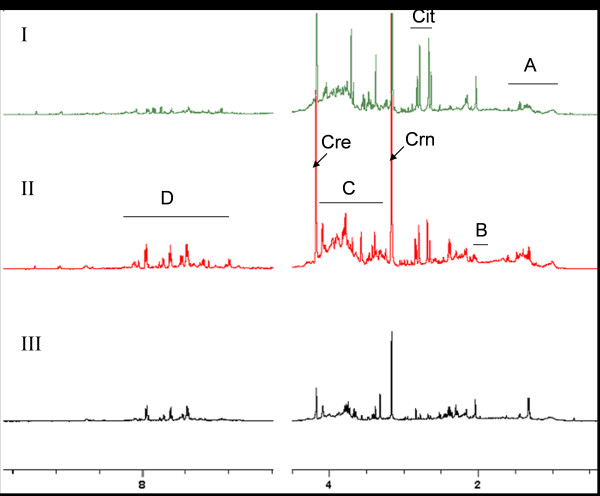
**Representative urine ^1^H NMR spectral profile of I**. Adult male patient with non-malarial fever, II. Adult male healthy individual and III. Adult male infected with *Plasmodium vivax*. However, due to high variability in the urine profiles of across individuals, a direct comparison of the spectral profile is not possible. Keys: A: Branched chain amino acid and small chain fatty acids, B: acetate and N-acetyl groups of acetylated amino acids, C: glucose, carbohydrates and amino acid alpha protons, D: Aromatic amino acids and metabolites, Cit: citrate, Crn: creatine/phosphocreatine/creatinine and Cre: creatine. The Urea and water regions were excluded from the figure.

^1^H NMR spectra of urine samples from patients and healthy individuals were recorded followed by multivariate statistical analyses. Representative ^1^H NMR spectra from individual belonging to the three groups are shown in Figure 1 [I-III]. These samples were from individuals infected with *P. vivax*, patients with non-malarial fever or healthy volunteers. For this analysis, only adult male samples were used. The individuals with infection had been referred to the pathology laboratories with malaria-like clinical symptoms (chills, fever, splenomegaly, etc.) and their infection status was confirmed by standard peripheral blood smears.

**Figure 2:**
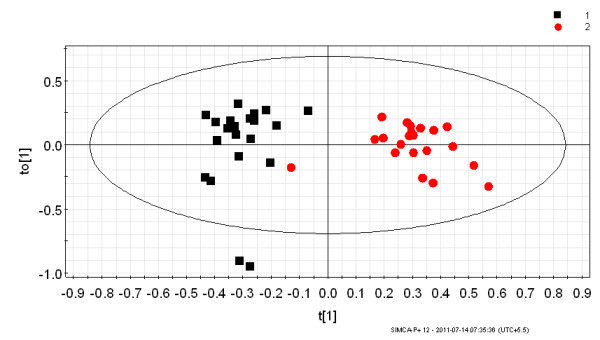
**OPLS-DA scores plot showing the variation between urinary metabolic profiles of the *Plasmodium vivax-*infected patients and healthy individuals**. This model was constructed from ^1^H NMR data of urine from 21 *P. vivax*-infected individuals and 21 healthy individuals. The subjects were all male. Each point in the plot represents one sample ^1^H NMR spectra. Black square = *P. vivax-*infected individual and red circle = healthy individual. The t[1] axis represents the predictive variation among the classes and the to[1] axis represents the variation orthogonal to the class specific variation. The statistical parameters of the model were as follows- R^2^X = 0.41 and Q^2^(cum) = 0.67. The ellipse is a 95% Hotelling's T^2 ^ellipse.

**Figure 3:**
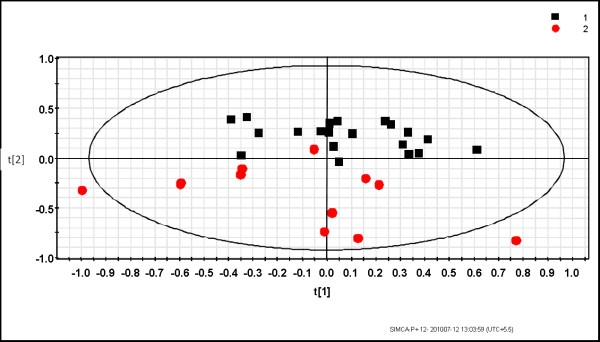
**PCA scores plot showing the variation between urinary metabolic profiles of *Plasmodium vivax-*infected individuals and non-malarial fever patients**. The PCA scores plot demonstrates that the infection by the parasite brings in considerable variation in the urine profile of the individuals. Here the variation is seen along PC2. This PCA model was constructed from ^1^H NMR of 21 patients with *P. vivax *infection and 12 patients with non-malarial fever. All of the patients were male. Each point represents one sample ^1^H NMR urine profile. Black square = *P. vivax-*infected individuals and red circles = non-malarial fever patients. R^2^X for the model = 0.67.

**Figure 4:**
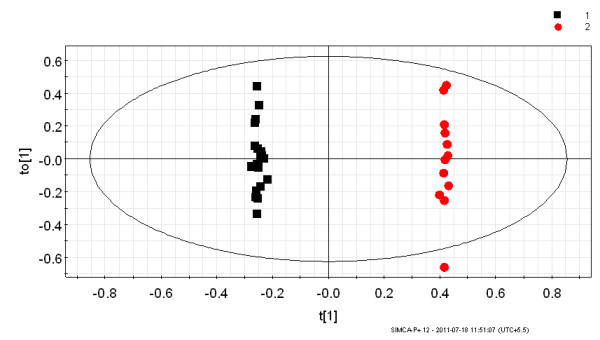
**OPLS-DA scores plot showing the variation between urinary metabolic profiles of *Plasmodium vivax-*infected individuals and non-malarial fever patients**. This model was constructed from ^1^H NMR data of urine from 21 *P. vivax*-infected individuals and 12 patients with non-malarial fever. All of the subjects were male. Each point in the plot represents one sample ^1^H NMR spectra. Black square = *P. vivax-*infected individual and red circle = non-malarial fever patients. The t[1] axis represents the predictive variation among the classes and the to[1] axis represents the variation orthogonal to the class specific variation. The statistical parameters of the model were as follows- R^2^X = 0.67 and Q^2^(cum) = 0.89. The ellipse is a 95% Hotelling's T^2 ^ellipse.

**Figure 5:**
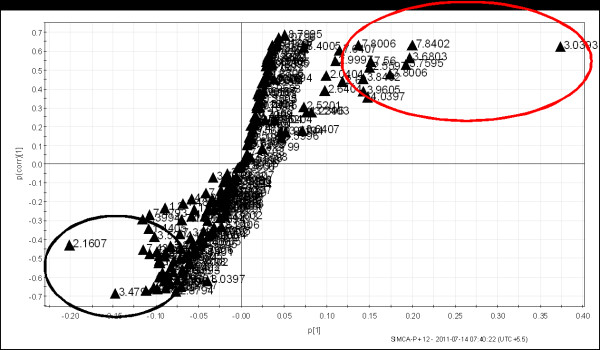
**A Representative OPLS-DA loadings S-plot showing relative contribution of bins/spectral variables to clustering of *Plasmodium vivax-*infected patients and healthy individuals**. This plot corresponds to Figure 2. Each point in the figure represents a bin. The p(corr)[1] axis represents the correlation of the bin towards the predictive variation shown in Figure 1. The p[1] axis represents the magnitude of the spectral bins. Bins from region circled in red represents the bins that decreased in the *P. vivax *infected individuals. Bins from region circled in black represents the bins that increased in the *P. vivax *infected individuals. Among them, bins with high p(corr)[1] (< -0.2 for increased and > 0.5 for decreased), high p[1] and VIP above cut-off (listed in Table 1) are selected for further analysis.

**Figure 6:**
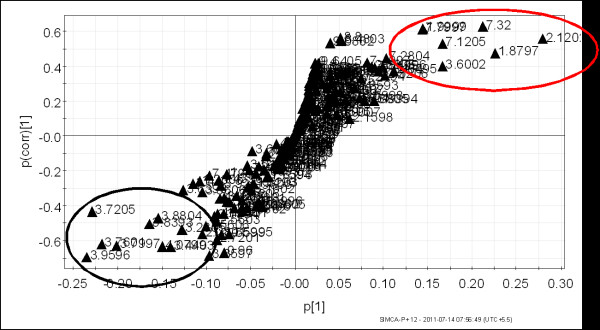
**A Representative OPLS-DA loadings S- plot showing relative contribution of bins/spectral variables to clustering of *Plasmodium vivax-*infected patients and non-malarial fever patients**. This plot corresponds to Figure 4. Each point in the figure represents a bin. The p(corr)[1] axis represents the correlation of the bin towards the predictive variation shown in Figure 1. The p[1] axis represents the magnitude of the spectral bins. Bins from region circled in red represents the bins that decreased in the *P. vivax-*infected individuals. Bins from region circled in black represents the bins that increased in the *P. vivax-*infected individuals. Among them, bins with high p(corr)[1] [< -0.4 for decreased and > 0.4 for increased], high p[1] (listed in Table 2) and VIP above cut-off are selected for further analysis.

The multivariate analysis was performed on two data sets obtained from the ^1^H NMR spectra of urine samples. The first data set consisted of urine from *P. vivax*-infected individuals and healthy controls. The second data set consisted of patients with malaria and non-malarial fever. Earlier data analysis by PCA of the ^1^H NMR spectra of urine from the healthy individuals and *P. vivax-*infected persons showed an inherent segregation between the two classes [20]. The R^2^X value was found to be 0.72 [20]. In the current study the OPLS-DA model was built to visualize the class specific segregation and to obtain the significant bins contributing to the variation across the classes, i.e. *P. vivax*-infected patients and healthy controls or non-malarial fever patients. The OPLS-DA scores plot from the urinary NMR metabolic profile of *P. vivax*-infected individuals and healthy controls is shown in Figure 2. This model also showed segregation of classes with R^2^X = 0.41 and Q^2^(cum) = 0.67. However, the spread is typical of human samples since there is enormous variation in genetic backgrounds as well as food habits. This is to say, the samples under consideration have factors other than malaria, which led to a larger scatter among them. To ensure that the effect of sex does not confound the analysis it was restricted to male patients only. In spite of all these factors, the Q^2^(cum) was found to be significantly high, therefore it implicated a considerable difference in the urinary metabolic profile of malarial patients and healthy individuals. OPLS-DA models made from NMR profile of urine samples of malaria patients and non malarial fever patients showed Q^2^(cum) = 0.89 and R^2^X = 0.67. This indicates a very good separation between the urinary profile of malaria patients and non-malarial fever patients, which is also evident from the PCA and OPLS-DA scores plot shown in Figures 3 and 4 respectively. In order to identify the spectral bins varying significantly between the classes, the loadings S-plot from the two models were analysed along with the Variable importance on Projection (VIP) values (Tables 1 and 2). The loadings S-plots are shown in Figure 5 (malarial patients and healthy individuals) and Figure 6 (malarial patients and non malarial fever patients). The bins accepted for further analysis are shown in Table 1 (malarial patients and healthy individuals) and Table 2 (malarial patients and non-malarial fever patients) along with their loadings and VIP values.

**Table 1:** OPLS-DA model bins selected for assignment of metabolites based on differences between patients of *vivax *malarial and healthy individuals

Increased/Decreased	Bin (Chemical shift)	Loading values	Variable importance on projection (VIP) values
Increased in urine metabolic profile of *P.vivax *infected patients	2.16	-0.20	3.09
	1.40	-0.12	2.11
	7.48	-0.11	2.04
	3.48	-0.15	2.10
	7.44	-0.12	1.79
	1.20	-0.09	1.58

Decreased in urine metabolic profile of *P.vivax *infected patients	3.04	0.37	5.61
	3.68	0.20	2.36
	3.80	0.17	2.29
	3.00	0.11	1.93
	2.04	0.10	1.51

**Table 2:** OPLS-DA model bins selected for assignment of metabolites based on differences between patients of *vivax *malarial and non-malarial fevers

Increased/decreased	Bin (Chemical shift)	Loadings values	Variable importance values
Decreased in urine metabolic profile of *P.vivax *infected patients	2.12	0.28	3.84
	1.88	0.23	3.10
	7.32	0.21	2.91

Increased in urine metabolic profile of *P.vivax *infected patients	3.96	-0.24	3.24
	3.72	-0.23	3.15
	3.76	-0.22	3.02
	3.80	-0.20	2.78

In order to identify the metabolites corresponding to the spectral bins found significant two-dimensional NMR techniques such as COSY and TOCSY were employed, matching of the chemical shifts with values reported in metabolite databases such as Human Metabolome Database (HMDB). The compounds so identified are listed in Tables 3 and 4, for the malaria *vs *healthy and malaria *vs *non-malaria fever patients, respectively.

**Table 3:** Urinary metabolites perturbed in the *Plasmodium vivax-*infected patients compared to healthy individuals.

Classes compared	Metabolites increased in *P. vivax*-infected individuals	Metabolites decreased in *P. vivax*-infected individuals
*P. vivax*-infected individuals and healthy controls	Valerylglycine	Creatine/Phosphocreatine
	N - acetylornithine	Tyrosine
	Salicylurate	Glucose
	pipecolic acid	Guanidoacetate
	Phenylpyruvate	Alanine
	biopterin, 3- hydroxybutyrate	N - acetylglutamate

**Figure 7:**
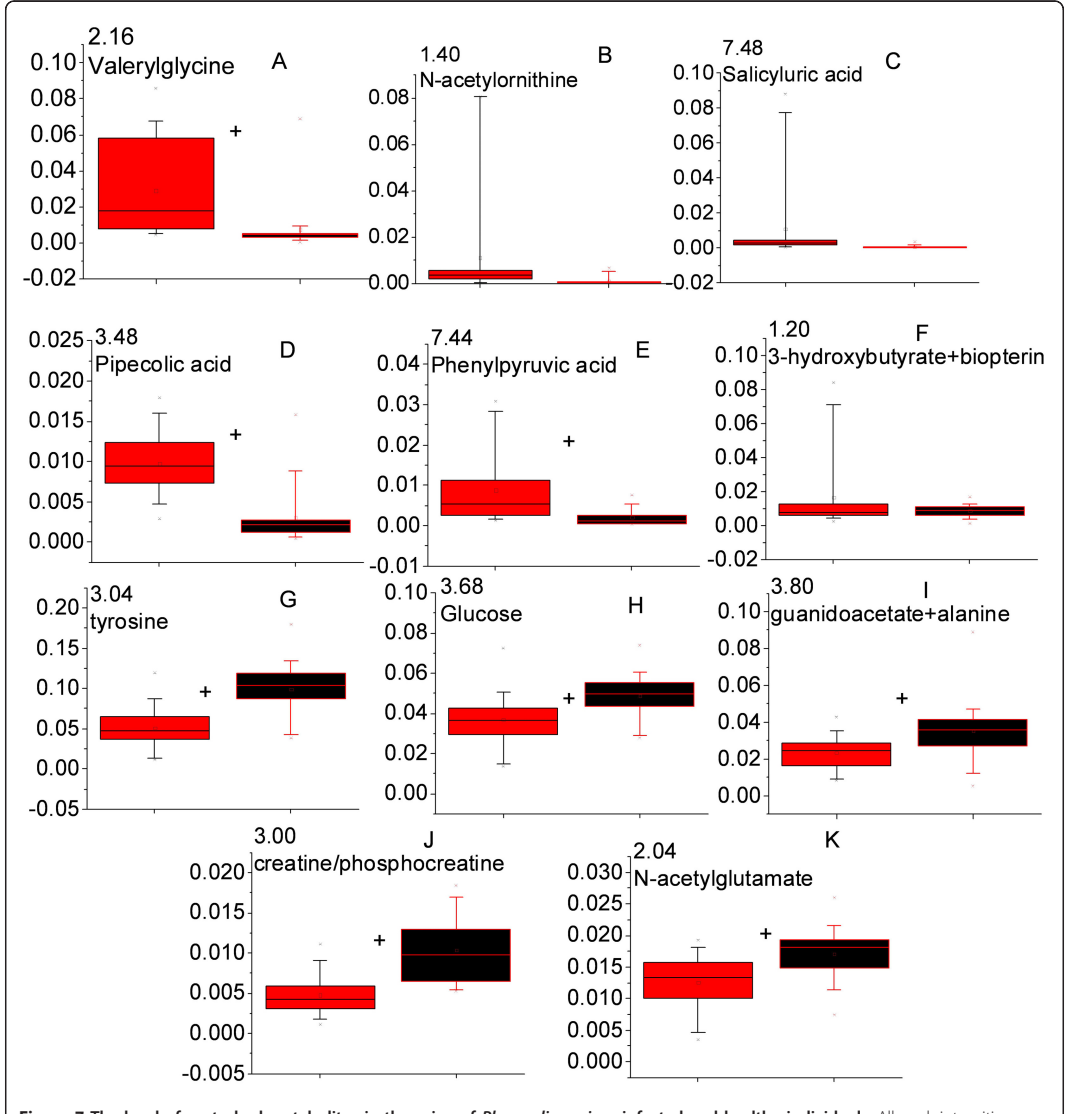
**The level of perturbed metabolites in the urine of *Plasmodium vivax*-infected and healthy individuals**. All peak intensities were calculated in terms of actual integrated value of individual NMR spectral peak normalized to the total spectral intensity. Panels with more than one metabolite represent an overlap in the peak of metabolites in the spectrum. Red = *P. vivax-*infected individuals and black = healthy individuals. + indicates *p *< 0.05.

Figure 7 indicates the bin corresponding to each metabolite

**Table 4:** Urinary metabolites perturbed in the *Plasmodium vivax-*infected patients compared to non-malarial fever patients.

Classes compared	Metabolites increased in *P. vivax*-infected individuals	Metabolites decreased in *P. vivax*-infected individuals
*P. vivax*-infected individuals and patients with non-malarial fever	L-phenylalanine	N-butyrate
	Hippurate	Acetate
	Glucose	
	Glutamine	
	Alanine	
	Ornithine	

**Figure 8:**
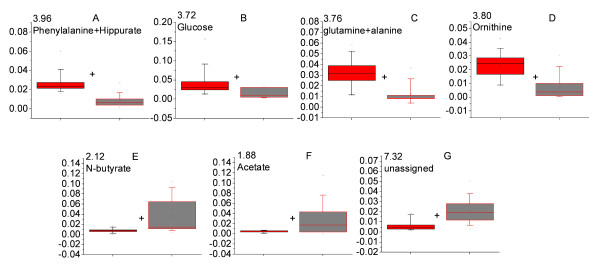
**The level of perturbed metabolites in the urine of *Plasmodium vivax-*infected and non-malarial fever patients**. All peak intensities were calculated in terms of actual integrated value of individual NMR spectral peak normalized to the total spectral intensity. Panels with more than one metabolite represent an overlap in the peak of metabolites in the spectrum. Red = *P. vivax-*infected individuals and gray = non-malarial fever patient. + indicates *p *< 0.05.

Figure 8 indicates the bin corresponding to each metabolite

### Integration of spectral peaks and univariate analysis

In order to compare the relative concentrations of the metabolites identified from OPLS-DA models, the corresponding ^1^H NMR spectral peak(s) were integrated. The results are presented in Figures 7 and 8 for the malaria patients compared with healthy individuals and patients with non-malaria fever respectively. Most of the metabolites showed significant variation across the classes. For example, the levels of valerylglycine, pipecolic acid, phenylpyruvic acid increased in the urine of malaria patients (Figure 7A, D, E) while the relative levels of tyrosine, glucose and N-acetylglutamate decreased in the malaria patients in comparison to that of healthy individuals (Figure 7G, H, K). Some spectral resonances had overlap from more than one metabolite (Figure 7F, I). These signals showed significant variation across the three groups. Since the contribution of individual metabolites towards such signals could not be ascertained, they are mentioned here collectively.

Several metabolites also showed significant variation across malaria and non-malarial fever patients. Among them, glucose and ornithine were increased in the urine profile of the malaria patients significantly (Figure 8B, D), while a significant decrease in N-butyrate and acetate levels was observed (Figure 8E, F).

**Figure 9:**
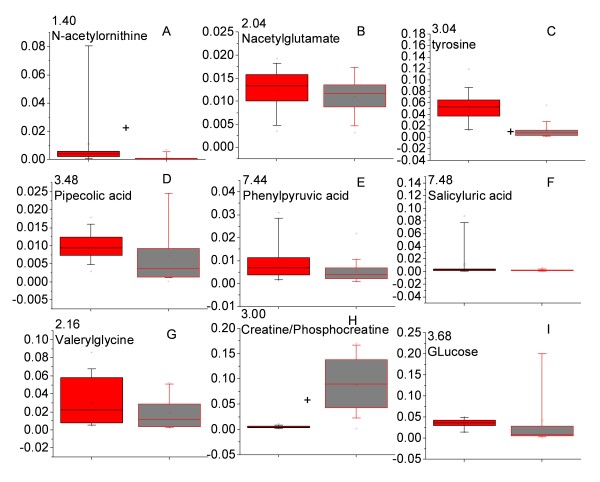
**The level of perturbed metabolites in the urine of *Plasmodium vivax*-infected and non-malarial fever patients**. Only those peaks are integrated which showed no overlap in the spectra and showed significant variation across the *P. vivax-*infected patients and healthy individuals. All peak intensities were calculated in terms of actual integrated value of individual NMR spectral peak normalized to the total spectral intensity. Red = *P. vivax-*infected individuals and grey = non-malarial fever patient. + indicates *p *< 0.05.

From Figures 7, 8 and Tables 3 and 4, it is evident that there is little overlap between the sets of metabolites found to be varying across the classes. Therefore metabolites showing differences between malaria patients and healthy individuals (Figure 7, Table 3) were integrated in the spectral profile of non-malarial fever patients and were compared with the malaria patients using univariate statistics (Figure 9A-I). Significant enhancement was observed in the concentration of N-acetylornithine and tyrosine (Figure 9A, C; *p *< 0.05 for both) in the malaria patients. Along with this, pipecolic acid showed a trend towards increase in the malaria patients compared to non-malarial fever, although this was not statistically significant (Figure 9D, p = 0.08).

## Discussion

*Plasmodium vivax *malaria is more prevalent than *P. falciparum *malaria in most parts of India [3]. Infection of *P. vivax *is historically believed to be a benign form of malaria. However recent reports suggest that *P. vivax *can also result in several complications including multiple organ failure, acute respiratory distress syndrome and CNS related problems [21-23]. It is, therefore, becoming even more important to understand the host-parasite interaction for vivax malaria, specifically how the host metabolism responds to the parasite infection. In this paper, a ^1^H NMR approach is described to understand this process. Urine metabolic profiles of *P. vivax-*infected individuals, healthy individuals, and patients with non-malarial fever were compared in order to get the malaria specific signatures.

Metabolic syndromes like lactic acidosis due to enhanced glycolysis are known complications during severe malaria [15,24]. However, enhancement in excretion of lactic acid was not observed in vivax-infected patients. This might be due to low parasitaemia (ranging from 0.01%-0.1%), resulting in insignificant perturbation of glucose metabolism. However, a range of other metabolites were found, which differentiate between the malarial patients and the two control categories.

The urine of malaria patients contained significantly increased amounts of valerylglycine and phenylpyruvic acid compared to that of healthy individuals (Figure 7A, E). However, the level of valerylglycine was not significantly different between the malarial patients and non-malarial fever patients (Figure 9G). Valerylglycine is a minor metabolite in fatty acid break down. The elevation of the urinary acylglycines is an indication of impaired β-oxidation of fatty acid [25]. Therefore, it may be concluded that the impairment in fatty acid β-oxidation is associated with febrile condition.

Data presented here also indicates a disturbance in the phenylalanine metabolism. Phenylalanine is known to be metabolized by phenylalaninehydroxylase and tetrahydrobiopterin (THBP) to tyrosine. Alteration of this pathway leads to the formation of phenylpyruvate. Usually phenylpyruvate is a minor metabolite of phenylalanine. However, in patients with phenylketonuria, the phenylalanine-tyrosine pathway is blocked leading to excess formation of phenylpyruvate [26]. A significantly enhanced excretion of phenylpyruvic acid in the malarial patients compared to healthy individuals was observed in our experiments (Figure 7E). This 'phenylketonuric condition' is probably arising out of an impaired phenylalanine metabolism. The data also suggests a possible increase in the biopterin concentration in the urine of malaria patients (Figure 7F). Some atypical form of phenylketonuria has been characterized by an increase in the urinary biopterin, which is caused by malfunction of dihydropteridinereductase (DHPR) [26]. Hyperphenylalaninemia is associated with severe *P. falciparum *malaria in African children [27]. However, this does not seem to be the case in the present study as the phenylpyruvic acid excretion is not significantly different between the malaria and non-malarial fever patients (Figure 9E). Although, the overlapped peaks of phenylalanine and hippurate showed a significant increase in the urine of malaria patients compared to that of non-malarial fever patients (Figure 8A), this cannot be taken to an indication of hyperphenylalaninemia. The hyperphenylalaninemic condition may be an associated complication of fever. However, there seems to be no study that deals with the metabolite profiling during fever in humans, therefore it remains an interesting aspect to be investigated.

Ornithine is a part of the urea cycle. In the first step of the cycle, ornithinecarbamoyltransferase (OCT) acts on ornithine and carbamoylphosphate to begin the natural detoxification of ammonia. Earlier reports suggested that elevation of serum value of OCT can be a potential marker of malaria and this was associated with the damage of the liver cells in the liver stage of the parasite [27,28].

Perturbation of OCT levels are likely to result in an impaired urea cycle. Urea cycle is the major pathway for ammonia detoxification in mammals. Earlier reports indicated ammonia toxicity during malaria in mice model [29]. Observation of significant increase in the urinary level of both ornithine (Figure 8D) and N-acetylornithine (Figure 9A) in malaria patients (compared to non-malarial fever patients) and an increased level of N-aceylornithine (compared to healthy individuals) (Figure 7B) also points to possible hepatic injury and impaired urea cycle resulting in ammonia toxicity during *P. vivax *infection. In the light of results presented here, earlier reports of statistically significant decrease of ornithine in the plasma of patients with clinical malaria [27] may be interpreted as an enhanced excretion of ornithine in malaria patients.

Pipecolic acid is a minor metabolite of lysine catabolism. In humans, pipecolic acid is found to be associated with chronic liver disease, pyridoxine dependent epilepsy and Zellweger syndrome [30-32]. Recent studies in the murine malaria model show elevated pipecolic acid level in the urine of NMRI mice [16]. Liver dysfunction is associated with malaria. However, the cause for the elevated pipecolic acid during malaria remains unclear. Although statistically not very significant, this metabolite was present in larger quantities in the urine of malarial patients compared with non-malarial fever patients (Figure 9D). Earlier studies showed hyperlysinaemia in the sera of *P. berghei *ANKA infected mice [15]. Hyperlysinaemia is often related to activation of minor metabolic pathway hence an enhanced pipecolic acid excretion [33]. From the data reported here and earlier data from murine malaria model it seems likely that lysine metabolism is perturbed during the course of malarial infection. However, the underlying biochemical reason remains to be addressed.

The non-malarial fever category serves as a control for general fever related changes that are not specific to *P. vivax *infection. Febrile illnesses are often likely to be misdiagnosed as malaria. Lundqvist and co-workers showed that borreliosis can be misdiagnosed as malaria due to the similarity in the symptoms [34]. Poorly executed microscopy also sometimes adds on to this, leading to malaria overdiagnosis and overuse of anti-malarial in malaria endemic regions [35,36]. In this report, it is shown that the non-malarial fevers can be separated from vivax patients on the basis of the urinary metabolite profile of the patients. The urine metabolomic profile seems to be significantly different in these two categories (Figures 3 and 4). This is due to the difference in the levels of Phenylalanine, hippurate, glucose, glutamine, alanine, and ornithine which are increased in the urine of the *P. vivax*-infected individuals and n-butyrate and acetate which are decreased in these patients (Figure 8, Table 4). Pipecolic acid also showed a higher trend in the urine of malaria patients compared to non-malarial fever patients (Figure 9D). As is evident from a very high Q^2^(cum) which is equal to 0.89 from the OPLS-DA model made from the urinary metabolite profile of the non-malarial fever patients and *P. vivax-*infected individuals, urinary metabolic profiles have a potential to be of diagnostic use for detecting malaria and to differentiate malaria from other non-malarial fevers.

## Conclusions

This study reports on the understanding of the host metabolic changes in terms of the urinary metabolite profile of the *P. vivax-*infected patients. Urinary metabolite profile of malaria patients were found to be distinct from both healthy individuals and those of non-malarial fever patients. Among several changes in metabolism, a disruption in the urea cycle in terms of ornithine excretion was noted, along with an elevated level of pipecolic acid indicating possible hepatic injury. Ornithine, therefore, promises to be candidate biomarker for malarial infection. Moreover, an extremely predictive model was constructed from urinary metabolite profile of the clinical malaria patients and individuals with non-malarial fever. This might help in the non-invasive diagnosis of malarial infection. Malaria-specific signatures were observed in the urine and showed that the urine profile of malaria patients cluster separately from different groups of control populations in spite of the high variability of urine metabolites across individuals. This study, therefore opens up the possibility of finding out malaria specific biomarkers in the urine, which might help in non invasive diagnosis of the infection in the long run. Many parameters, such as genetic make up, food habits and lifestyle, may influence urine metabolite patterns. In order to circumvent such confounding parameters, a longitudinal follow up and comparison of urine from cohorts of malaria patients and other control subjects will provide further specific information regarding disease related changes in patients of *P. vivax *malaria.

## Competing interests

The authors declare that they have no competing interests.

## Authors' contributions

AS, SG, SM, PJ were involved in collection and processing of the samples, NMR experiments, statistical analyses and model building and interpretation of the data. AS and SG, in addition, drafted the manuscript. SP, SS and HMS conceived the study, interpreted the data and were involved in writing the manuscript. All authors read and approved the final manuscript.

## Acknowledgements

The authors are grateful to the National Facility for High-field NMR, TIFR and Dr R.Y. Agarkar of TIFR Medical section for all their help. We thank Dr S. Bhayani and Dr A. Mehta for help in sample collection. AS thanks the Council of Scientific and Industrial Research, Govt. of India for the Shyama Prasad Mukherjee Fellowship.
